# Direct Imaging of Nanoscale Ferroelectric Domains and Polarization Reversal in Ferroelectric Capacitors

**DOI:** 10.1021/acs.nanolett.5c05032

**Published:** 2025-11-03

**Authors:** Megan O. Hill Landberg, Bixin Yan, Huaiyu Chen, Ipek Efe, Morgan Trassin, Jesper Wallentin

**Affiliations:** † MAX IV Laboratory, 5193 Lund University, 22100 Lund, Sweden; ‡ Department of Materials, ETH Zurich, 8049 Zurich, Switzerland; § Synchrotron Radiation Research and NanoLund, Department of Physics, Lund University, 22100 Lund, Sweden

**Keywords:** multiferroics, X-ray microscopy, nanodiffraction, ferroelectric devices, piezo-response force microscopy, ferroelectric domains

## Abstract

Ferroelectric thin films present a powerful platform for next-generation computing and memory applications. However, domain morphology and dynamics in buried ferroelectric stacks have remained underexplored, despite their importance for real device performance. Here, nanoprobe X-ray diffraction (nano-XRD) is used to image ferroelectric domains inside BiFeO_3_-based capacitors, revealing local disorder in domain architecture and partial polarization reorientation caused by the capacitor electrostatic boundary conditions and internal stress. We demonstrate sensitivity to ferroelectric reversal in poled capacitors, highlighting expansive/compressive (001) strain for up-/down-polarization using nano-XRD. We observe significant quantitative and qualitative differences between poling by piezoresponse force microscopy and in devices. Further, electrical poling induces lattice tilt at electrode edges, which may modify performance in downscaled devices. Our results establish nano-XRD as a noninvasive probe of buried ferroelectric domain morphologies and dynamics, opening avenues for operando characterization of energy-efficient nanoscale devices.

## Graphical abstract

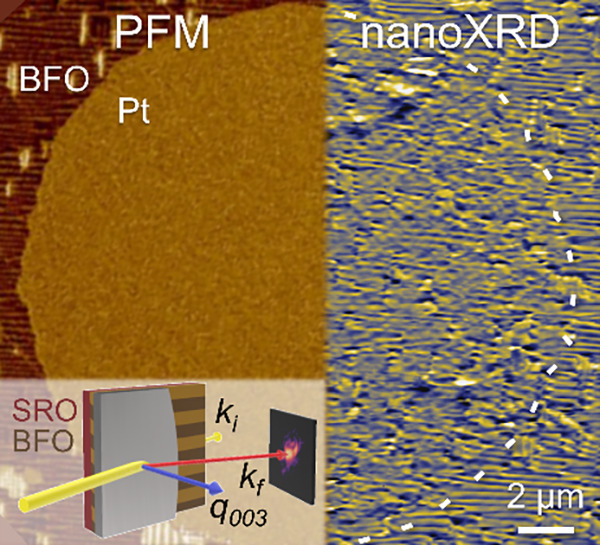

The study of ferroelectric thin films has been revived by the recent push for beyond-CMOS applications and low energy consuming emerging computing and memory schemes.^1,2^ Such devices, like ferroelectric random-access memory, rely critically on robust polarization reversal to achieve sufficient retention and fatigue properties.^3^ Various mechanisms of polarization fatigue have been explored in traditional perovskite ferroelectrics which include polarization pinning by oxygen vacancies or domain walls.^4,5^ Particularly important to device performance is the nanoscale domain architecture, as domain walls can act as sites for defect accumulation and conductive pathways that can lead to leakage.^6^ Recent studies highlight the critical need for imaging of domain structures to understand ferroelectric device performance.^7^ Control of ferroic domain architecture is also critically important for the design of next-generation logic and memory such as magnetoelectric spin–orbit or ferroelectric spin–orbit devices.^8,9^

However, imaging ferroelectric domains in real devices (i.e., buried configurations) with high resolution remains challenging using conventional techniques such as scanning probe, electron, or optical microscopy, since the top electrode of capacitors covers the ferroelectric film.^10,11^ Therefore, the impact of device integration on ferroelectric domain architecture and the study of their dynamics upon poling at the nanoscale remain underexplored. Advances in X-ray sources and optics have allowed nanoprobe X-ray diffraction (nano-XRD) to reach below 100 nm spatial resolution while maintaining long penetration depths. Researchers have imaged the dissolution of vertical domain walls (DWs) under in-plane electric fields,^12^ imaged domain evolution under photoexcitation,^13^ and investigated biasing of ferroelectric devices,^14,15^ but experimental constraints have mostly limited domain structures from being spatially resolved.

Here, we use nano-XRD to image nanoscale domains within ferroelectric capacitor structures, providing newfound clarity into ferroelectric domain ordering under metallic electrodes and upon polarization switching. Taking ferroelectric BiFeO_3_ (BFO) as our model system, we resolve nanoscale domains directly within capacitors and demonstrate X-ray sensitivity to polarization switching both in bare films and under thick electrodes. We reveal that mere deposition of metal contacts induces a domain pattern modification, prior to any device poling. At the edges of capacitors, we observe strikingly different domain response to poling with implications for polarization switching upon downscaling. While revealing unexpected structure changes in BFO devices, this work also highlights the potential for nano-XRD as a powerful tool to investigate a wide range of ferroelectric devices.

We first studied domain patterns in as-deposited ferroelectric thin films by comparing nano-XRD and conventional piezoresponse force microscopy (PFM). Motivated by recent reporting on ultralow energy consuming logic devices by integration of ferroelectric magnetoelectric BFO, we grew BFO (50 nm) on SrRuO_3_-buffered (SRO) DyScO_3_ (DSO).^16^ Rhombohedral (*R*3*c*) BFO presents four ferroelastic domain variants with polarization pointing along one of four ⟨111⟩ pseudocubic (PC) directions. The ∼0.4% compressive epitaxial strain produces a ferroelectric domain architecture consisting of stripe-like 71° ferroelastic domains along DSO [001].^17,18^ Platinum electrodes were then deposited on the BFO/SRO/DSO to finalize the metal–ferroelectric–metal capacitor heterostructures. Figure 1a shows the striped domain structure with respect to the DSO and BFO crystallographic axes. Nano-XRD was performed in the geometry shown in Figure 1b, collecting high-resolution 2D diffraction patterns of the (003)_PC_ peak (Figure 1c) at each probe position. The center of mass of the Bragg peak was calculated for each probe position, producing tilt and strain maps. More details about sample growth and diffraction measurements can be found in Supplementary Note 7.

**Figure 1:**
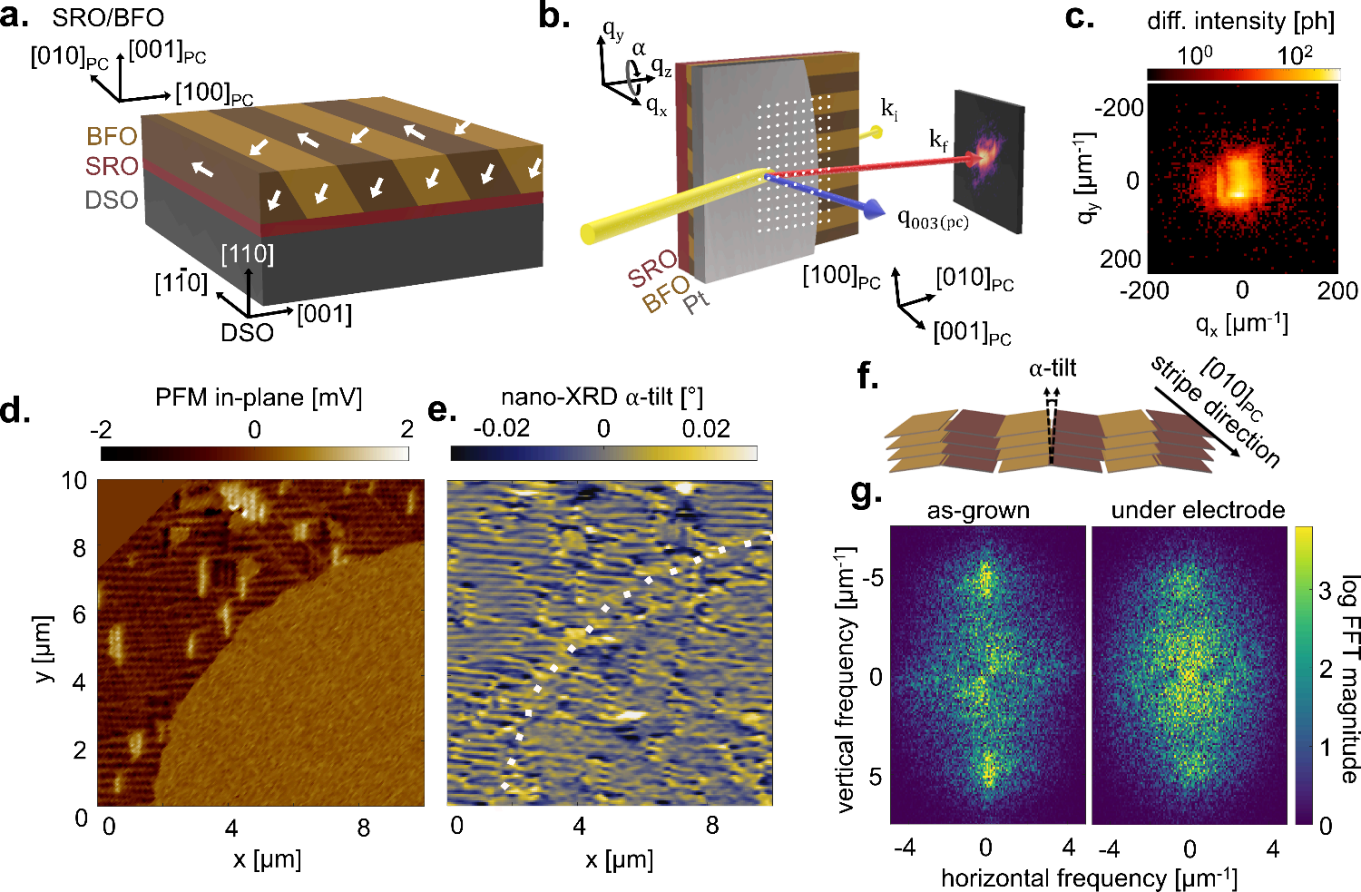
(a) Schematic of the BFO stack with AG ferroelectric domain directions marked in white. Axes given for the DSO crystallographic directions and the SRO/BFO_PC_ directions. (b) Nano-XRD scattering geometry for the capacitor stack with the X-ray probe in yellow, *q*-vector in blue, and *k*_f_ in red. Tilt direction α, described by the rotation around *q*_*z*_. Example probe positions marked in white. (c) Exemplary diffraction pattern of BFO (003)_PC_ for the AG region of the film. (d) In-plane PFM map of unpoled electrode e:0V (∼1/4 of electrode shown). (e) Nano-XRD α-tilt map for the same electrode. Dotted white line shows the outline of the contact extracted from PFM. (f) Schematic describing the tilt relationship of α with the domain stripe direction. (g) 2D FFT magnitude of AG (left) and contacted (right) regions of BFO from Figure S3b.

First, using an unpoled BFO capacitor (“e:0V”), we compare PFM and nano-XRD demonstrating that the nanoscale BFO domain architecture, here ∼100 nm wide stripe-like ferroelastic domains, can be spatially resolved using both techniques. The strength of nano-XRD, however, strikingly appears when investigating the nanoscale domain structure buried under 100 nm of Pt. While the thick metal electrode impacts the local electric field line distribution and hence deteriorates the PFM spatial resolution, nano-XRD imaging remains unaffected (side-by-side comparison in Figure 1d,e). Nano-XRD generates several types of contrast, but here we show the α-tilt, which refers to rotation around *q*_*z*_ (defined in Figure 1b). This is approximately the tilt of (001) planes *between* ferroelectric domain stripes (along [100]_PC_), as schematized in Figure 1f. Indeed, a ferroelastic tilt is expected between 71° domain stripes. For statistical analysis, a larger map was taken in the vicinity of another contacted BFO region (Supplementary Note 2). 2D FFT analysis generates log-power spectral density maps (Figure 1f) for a 135 μm^2^ area from an as-grown (AG) region (left) and a region buried under the electrode (right). The AG region shows clear vertical lobes at about ±5 μm^–1^, indicative of highly aligned horizontal stripe domains with ∼100 nm domain width. The contacted region, on the other hand, shows a much more diffuse FFT, pointing to a more irregular domain structure. Due to this disorder, the distribution of tilts is also slightly lower for the contacted region (Figure S3d).

Next, nano-XRD was used to probe the polarization reversal in electrically switched capacitors. Three electrodes were poled *ex situ* to induce positive polarizations with out-of-plane polarization components toward Pt; for details see Supplementary Note 7. Figure 2 compares poled e:6V (p-up) with the pristine device e:0V (p-down) using three types of contrast taken from the same nano-XRD maps. The α-tilt images reveal a stark and surprising difference in domain response between the edge and the device interior (Figure 2a,b). In the device interior we see little change due to poling. Indeed, when plotting an α-tilt histogram (Figure 2e, top), there is no variation across the four electrodes tested, indicating that the average domain structure does not change significantly upon poling. In contrast to average tilt distribution, we observe a clear increase in α-tilt around the contact edge of the poled device (e:6V) shown in Figure 2a, bottom. This edge effect is present but barely visible in the pristine device (e:0V), Figure 2a, top. This tilt is observed along most of the poled device edge, but is not consistent across the whole device, possibly related to local variations in electrode deposition. The edge effect should be considered as a potentially significant contribution to device behavior, since strain and tilt are closely intertwined with polarization. A careful comparison shows that the affected region extends from the contact edge to about 0.75 μm inside, making it invisible to PFM. In our large prototype devices (10 μm radius) the high-tilt ring is already ∼14% of the device area. For more typical device scales (∼1 μm radius) this high-tilt ring would make up >90% of the overall area, dominating device behavior. We note that modification of the nanoscale polarization configuration takes place solely within the rim of the capacitor underneath the top electrode. Despite the continuous nature of the SrRuO_3_ bottom electrode, there is no evidence of stray fields outside the capacitor. This points to the limited charge-screening efficiency and stress-induced effects of the top electrode. Our study suggests that the scaling-down limitations caused by internal stress and electrostatic boundary condition degradation should be carefully considered even when fully etched ferroelectric capacitor islands are employed.

**Figure 2:**
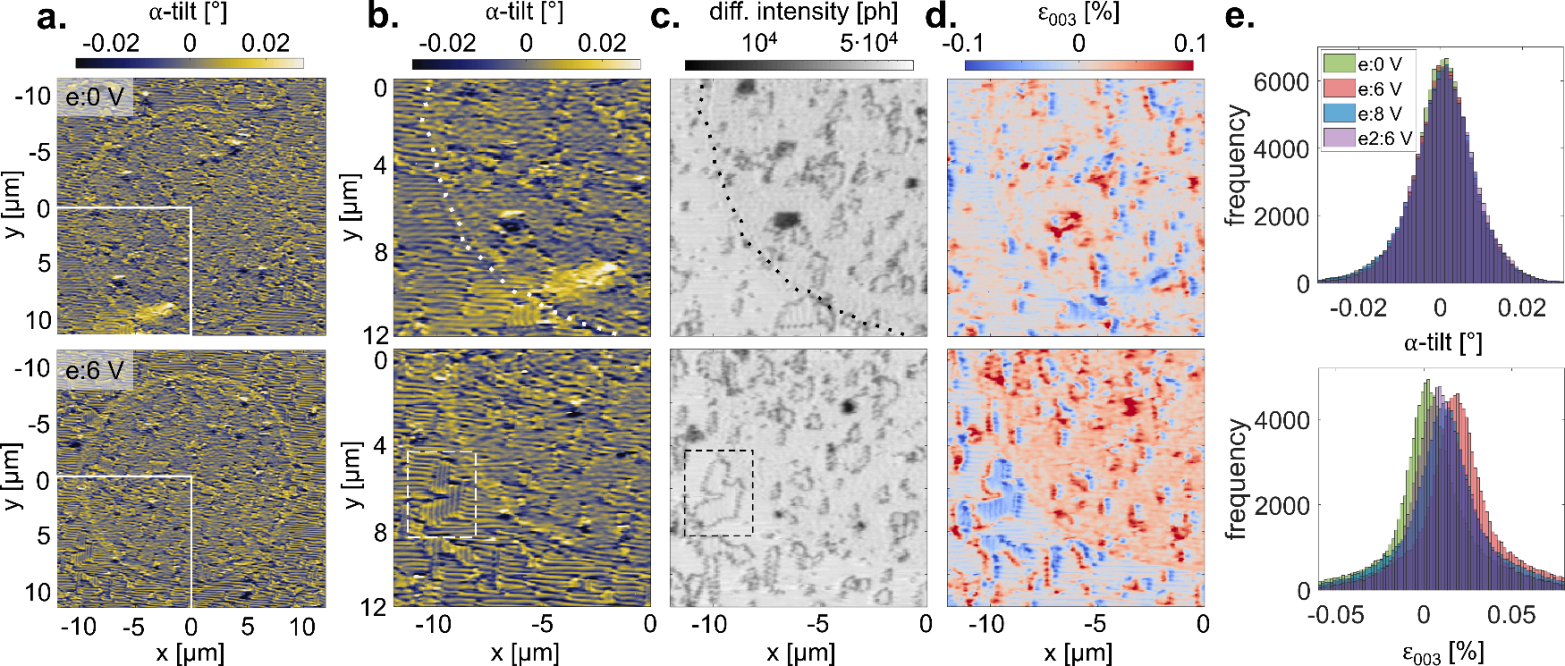
(a) Nano-XRD maps of α-tilt for e:0V on top and e:6V on bottom. The region marked with a white square is extracted in (c, d). (b) Zoomed-in maps of (b) α-tilt, (c) diffraction intensity, and (d) estimated ε_003_ strain for e:0V (top) and e:6V (bottom). Dotted white/black lines show the outline of the contact pad extracted from PFM maps. Dotted lines are not shown for maps where it would disrupt contrast. Dashed white and black boxes highlight an example region where vertical and horizontal domain stripes meet (109° domain walls). (e) α-tilt distribution (top) and strain distribution (bottom) histograms for all positions under four electrodes: e:0V is pristine (p-down) and e:6V, e:8V, and e2:6V are switched (p-up).

Figure 2c shows the diffraction intensities for e:0V (top) and e:6V (bottom). While there is local variation in diffraction intensity from disorganized domain regions, there is no significant difference in intensity of BFO under electrodes versus in the AG regions for either poled or pristine devices. This invariance in diffraction intensity means that estimated (003) strain can be extracted from a single diffraction angle (see Supplementary Notes 1, 3). Figure 2d shows ε_003_ strain for e:0V (top) and e:6V (bottom). Comparing the global behavior of strain under electrodes versus AG regions, we see mostly invariant strain for e:0V. However, e:6V shows a measurable increase in strain under the electrode compared to the AG region. This suggests that poling the capacitor to p-up induces an out-of-plane lattice expansion. Indeed, this is seen for all poled electrodes, as shown by the histogram in Figure 2e, bottom. Here, the ε_003_ strain distribution, normalized by the surrounding AG region, is shown for all four contacts. While the mean strain is different for each electrode, all poled electrodes (e:6V, e:8V, e2:6V) show a strain increase. The mean strain difference between p-down (e:0V) and p-up (e:6V) is 0.025%. Nano-XRD maps for all other devices are in Supplementary Note 4.

Another notable feature is present where horizontal and vertical domains meet, for example, the dashed box in Figure 2b,c, which in BFO constitutes 109° DWs. Interestingly, at this interface, diffraction intensity decreases (Figure 2c), which is not seen at the 71° DWs (see Supplementary Note 5). The 109° DWs are parallel to the scattering vector (along [001]_PC_), which may produce more pronounced sensitivity to structural changes, despite their small size (1–5 nm). For instance, regardless of DW type (Ising, Bloch, Néel),^19^ out-of-plane polarization will be zero at the DW, inducing localized electrostriction in the [001]_PC_ direction. It has been previously observed that even purely ferroelectric (180°) DWs are mechanically unique from their surroundings.^20^ Though DWs are not resolvable with the 60 nm probe, they can interfere with the coherent beam, reducing local scattering intensity. This sensitivity suggests that X-ray nanoprobes will be useful for investigating the structure of DWs in ferroelectrics.

Finally, for detailed comparison of the sensitivity of nano-XRD to polarization switching, we imaged the bare film around a box-in-box structure written by PFM (outer box: −5 V, inner box: +5 V). Out-of-plane (OOP) and in-plane (IP) PFM measurements confirm the expected ferroelastic and ferroelectric switching behavior with the presence of 71°, 109°, and 180° DWs (Figure 3c). Diffraction measurements in this region were taken in two geometries, because these were identified to provide different contrast: (1) beam parallel to the ferroelastic domain stripes (Figure 3a) and (2) sample rotated 90° so that the beam is perpendicular to the domain stripes (Figure 3b). The beam footprint is illustrated by yellow ellipses. Nano-XRD produces contrast comparable to that measured by PFM even though the methods are of an entirely different nature.

**Figure 3:**
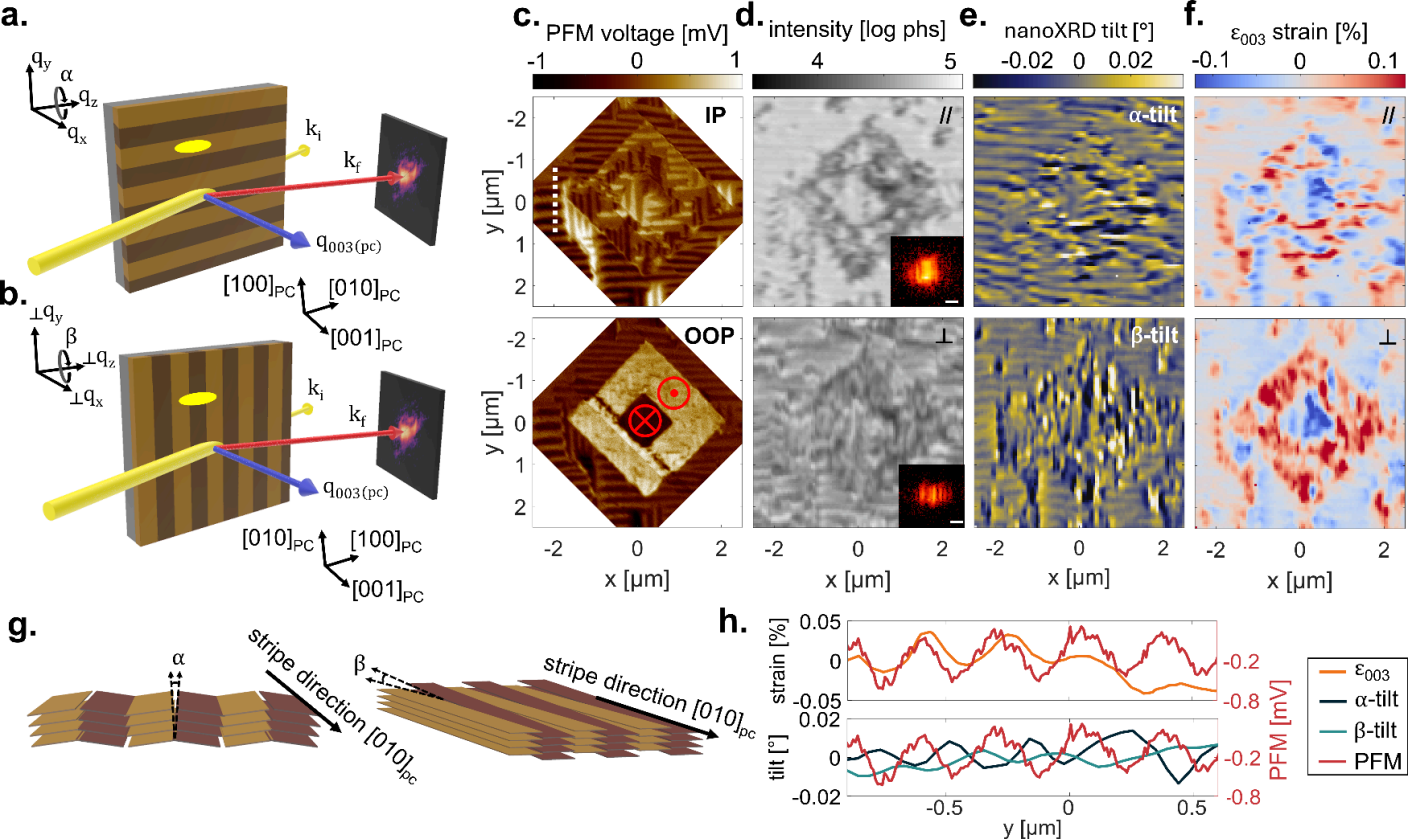
Scattering geometry for the probe parallel (a) and perpendicular (b) to the domains. X-ray probe is shown in yellow, *q*-vector in blue, and *k*_f_ in red. Approximate beam footprint with respect to the domains is shown by a yellow ellipse. (c) PFM maps of in-plane (top) and out-of-plane (bottom) polarization within the box-in-box region of the BFO, written by PFM. Red markers indicate polarization directions that were induced by PFM poling of ±5 V. (d) Diffraction intensity maps from parallel (top) and perpendicular (bottom) scattering of the same box-in-box region as in (c). Insets show exemplary diffraction pattern from the AG regions. Scale bars are 25 μm^–1^. (e) Nano-XRD tilt maps for α-tilt and β-tilt, defined by rotation around *q*_*z*_ for parallel (a) and perpendicular (b) scattering, respectively. (f) Estimated strain (ε_003_) maps from parallel (top) and perpendicular (bottom) scattering. (g) Schematic describing the tilt relationship α- and β-tilt with respect to the domain stripe direction. (h) Line cut extracted from the white dashed line in (c) comparing the in-plane PFM voltage with ε_003_ (top) and α-/β-tilt (bottom).

We first compare the simplest contrast type: diffraction intensity. Figure 3d shows parallel (top) and perpendicular (bottom) integrated diffraction intensity maps for the box-in-box region. Example diffraction patterns (insets) show notable differences, with the perpendicular geometry (bottom) showing strong interference fringes spaced ∼30 μm^–1^ in *q*_*x*_, consistent with repeating ferroelastic stripes with a width of ∼105 nm. This interference decreases the overall scattering intensity of the perpendicular compared to parallel geometry, even for the same region. This measurement compares to those in which multiple domains are probed simultaneously.

The box-in-box region is easily identifiable in the parallel scattering diffraction intensity with a lower intensity in the p-up region. Under first consideration, this suggests that in the p-up region, the Bragg angle is not met. However, a rocking curve in this region (Supplementary Note 5, Figure S6) shows that the intensity reduction is actually from interference, like how interference effects reduce scattering intensity in the perpendicular geometry. This may be due to interference from a high density of 109° and 180° DWs, as discussed previously. In the perpendicular geometry, intensity variation in the p-up region is less pronounced, possibly because the beam is illuminating multiple DWs, averaging out their effect. It follows that we do not see these interference effects in the poled capacitors, as the density of DWs is significantly lower, only at electrode edges.

As the Bragg condition is not significantly changing between poled regions (Supplementary Note 3), we can estimate tilt and strain within these areas despite having single angle measurements. Figure 3e shows α-tilt (top) and β-tilt (bottom), defined by rotation around *q*_*z*_ for parallel and perpendicular geometries, respectively. As schematized in Figure 3g, α-tilt probes tilt *between* ferroelectric domains (along [100]_PC_) and β-tilt probes tilt *along* domains (along [010]_PC_). In the AG region, ferroelectric domains are resolvable, with better resolution for α-tilt compared to β-tilt due to the smaller beam footprint. Within the poled region, given the interference effects, tilt is hard to quantify, but a striped structure matching PFM is still visible.

Figure 3f shows the strains for parallel (top) and perpendicular (bottom) geometries. These maps are sensitive to the same feature, ε_003_ strain, but the parallel geometry has a higher spatial resolution along the stripes due to the beam footprint. In both cases, we observe strain contrast from the box-in-box structure. This is more prominent for the perpendicular geometry due to averaging effects across multiple domains, resulting in the largest contrast for polarization changes. Thus, we find that the most striking effect measured with nano-XRD is an out-of-plane strain with compression for p-down (−0.06%) and expansion for p-up (+0.06%). This is similar to the observation for poled capacitors, though the average strain magnitude is higher for PFM poling compared to electrode poling, which exhibits only a 0.025% strain difference between p-down and p-up electrodes.

It is difficult to determine the precise nature of the strain observed here. A mixture of effects is expected, given the presence of multiple domain types within the PFM poled region. The high density of 109° and 180° DWs is likely to produce strain variations like those in the AG region where 109° DWs are present, where vertical and horizontal domain stripes meet. These domains could produce higher strain values measured in Figure 3f, giving an artificially higher average strain change with polarization switching as compared to poled capacitors.

Finally, for direct correlation with polarization, Figure 3h shows a line cut from the white dashed line in Figure 3c. We observe that strain varies with the same period as the domain stripes, unsurprising given the lattice mismatch of ferroelastic domain interfaces. Tilt shows the same trend with α-tilt oscillating in the same period as the polarization. However, interestingly, the β-tilt, which is sensitive to tilt *along* domains, is offset about half a period from the domain stripes. While α-tilt is sensitive to ferroelastic tilt, it is possible that this offset in β-tilt could be related to the known chiral structure at the 71° DWs in BFO.^21,22^ Further maps of the AG region are shown in Supplementary Note 1, where α-tilt, β-tilt, and strain are more precisely quantified by analyzing angular rocking data.

We establish that deposition of metal electrodes alters the domain structure in ferroelectric thin films. This makes clear that assumptions about buried films, taken from imaging AG regions, do not hold, as such changes in the domain structure can modify the overall performance. The observation that the overall tilt distribution in buried BFO is lower than that in the AG film has implications for device integration of such multiferroic materials. This may be related to metal stress, which is a well-known phenomenon in microelectronics,^23,24^ modifying the domains by elastic coupling in BFO.^25^ It is also possible that electrostatic boundary conditions, i.e., screening of the surface bound charge, are modified following the top electrode deposition, further influencing polarization configuration in our films. Electrode deposition might, for instance, result in transient enhancement of depolarizing field contributions,^26^ as charge screening surface adsorbates spontaneously formed after the material deposition^27–29^ may be sputtered off during additional processing.

Further, we show quantitative and qualitative differences in domain dynamics for switching by PFM and electrodes. We compare the domain structure of pristine and poled capacitors: poling induces a tilt around the contact edge, which could significantly impact the behavior of smaller devices. Beyond modification of the domain structure, this localized tilt will produce a nanoscale strain gradient which may drive oxygen vacancy redistribution upon cycling, detrimental to fatigue.^30,31^ No similar effect is observed for PFM-poled regions, suggesting that this tilt is directly related to the presence of an electrode.

We identify lattice spacing changes upon poling in both capacitors and PFM-poled films. Strain variations between p-up and p-down appear substantially larger for PFM poled films as compared to capacitors, likely due to high densities of 180° and 109° domain walls in the box-in-box structure, producing artificially higher strain magnitudes. However, the overall trend in both PFM-poled and capacitor regions suggests that polarizing BFO films produce an out-of-plane [001] *d*-spacing change: expansive for p-up and compressive for p-down. This is surprising, as pure 180° polarization reversal should only displace Bi atoms. However, it is clearly observed here that polarization switching is coupled to a structural change. A similar effect was observed previously by Jo et al. in Pb­(Zr,Ti)­O_3_, with 0.1% compressive strain observed in p-down written regions, which authors attribute to insufficient polarization screening.^32^ However, in our case, if this effect was the result of a built-in field, we would expect a strain modification even for the nonpoled electrode (Figure 2d, top). Instead, we hypothesize that this unidentified structural coupling may be related to ion reorganization, as observed previously in BFO even for low fields.^33^ Note that this is a very small and localized strain effect on the scale of 0.02% to which other characterization techniques, including AFM, are insensitive. This structural change warrants additional study, as it could exacerbate polarization fatigue present for 180° switching by making domains further susceptible to pinning under increasing internal stresses.^34,35^ This is particularly important for scaled devices, as additional strain will modify the local energy landscape and possibly (de)­stabilize smaller ferroelectric domains, reducing device retention. Further, such local strain gradients may exacerbate defect accumulation, reducing device endurance.^30,32^

Finally, we demonstrate that nano-XRD is highly sensitive to 71° ferroelastic domains and even DWs via interference effects. Quantifying such interference effects may be possible via other X-ray techniques, such as Bragg ptychography, pointing to opportunities for X-rays to investigate or even image DWs.

In summary, polarization reversal in BFO produces complex structural changes that could affect the reliability and cyclability of ferroelectric devices. However, further insights from imaging buried films or studying domain dynamics through operando-compatible techniques like nanofocused X-ray imaging can increase our understanding of device performance. This may allow for better tailoring of electrode depositions to minimize domain reconfiguration or even strain engineering of ferroelectric domains through the controlled top-layer deposition. Ultimately, understanding domain morphology and dynamics within buried ferroelectric stacks, which can differ substantially from bare films, will improve our ability to control and design next-generation computing and memory devices.

## Supplementary Material



## Notes

Details on 3D strain mapping analysis (Note 1) including image analysis using all rocking angles (Figure S1) and example rocking curves (Figure S2); details on statistical analysis of tilt maps (Note 2); discussion on determining strain without full rocking curves (Note 3); full maps of all biased electrodes (Note 4); rocking curves showing interference effects (Note 5); images of additional box-in-box structure (Figure S7) and beam damage testing (Figure S8); supplementary methods on XRD measurements, sample fabrication, electrical poling, and PFM measurements (Note 7) (PDF)
