# Supplementary material for: Direct Imaging of Nanoscale Ferroelectric Domains and Polarization Reversal in Ferroelectric Capacitors
Source: Nano Lett. 2025 Nov 3;25(45):16304–10. doi: 10.1021/acs.nanolett.5c05032 (PMC12616765; doi:10.1021/acs.nanolett.5c05032)
Supplement: Supplementary file 1 [file nl5c05032_si_001.pdf]

Supplementary Information: Direct imaging of nanoscale ferroelectric domains and polarization reversal in ferroelectric capacitors

Megan O. Hill Landberg[1*], Bixin Yan[2], Huaiyu Chen[3], Ipek Efe[2], Morgan Trassin[2], and Jesper Wallentin[3]

[1] MAX IV Laboratory, Lund University, 22100 Lund, Sweden
[2] Department of Materials, ETH Zurich, 8049 Zurich, Switzerland
[3] Synchrotron Radiation Research and NanoLund, Department of Physics, Lund University, 22100 Lund, Sweden
*Corresponding author: megan.landberg@maxiv.lu.se

Supplemental Note 1: 3D strain mapping analysis.

3D strain mapping was performed on an as-grown (AG) region of the film in the parallel scattering geometry. Scans were repeated at 6 angles (0.02° step size) to guarantee that the Bragg condition was fulfilled for the majority of scan positions, as seen in the integrated diffraction intensity map of a region within the AG film (Figure S1a). An example diffraction pattern for the AG region is shown in Figure S2a, the location of this pattern is marked by a red dot in Figure S2b. A rocking curve in the vicinity of this red dot is shown in Figure S2c. Example diffraction patterns along this rocking curve are also shown in Figure S2d.

Strictly speaking, precise strain and tilt mapping requires collection of 3D diffraction images as performed for this region. To quantify tilt and strain, first real space pixels, $\Delta_{x,y}$ on the detector image must be converted to reciprocal space units, dq_1 and dq_2, calculated as follows: $dq_{1,2} = \frac{2\pi\, \Delta_{x,y}}{D\lambda}$, where D is the detector distance from the sample and λ is the wavelength. The third dimension is defined by the angular steps taken (sample rotation steps $d\theta$): $dq_3 = \frac{2\pi\, d\theta \sin(\theta)}{\lambda}$ where θ is the Bragg angle. The reason that 3D diffraction patterns are required, is that the coordinates q_1, q_2, q_3 are not orthogonal, but q_1 and q_3 are dependent on each other. To convert to an orthogonal coordinate system, the following transformations were performed: $q_x = q_1 \cos(\theta)$; $q_y = q_2$; $q_z = q_3 - \sin(\theta)$.

Quantification of (003)pc strain and tilt were performed by extracting the peak centre-of-mass (COM) in q_x, q_y, and q_z. Given the high flux of the diffraction patterns (>10^5 integrated photons at each probe position) the COM can be resolved with sub-pixel resolution providing a tilt resolution better than 0.0044°. Relative tilts are calculated by measuring the deviation in the Bragg peak COM and converting to rotations around q_z (α-tilt) and q_y (β-tilt) as follows: $\alpha = \sin^{-1}\frac{q_y}{|Q|}$ and $\beta = \tan^{-1}\frac{q_x}{q_z}$, $|Q|$ is the modulus of all COM components. The d-spacing is defined by the modulus: $d = \frac{2\pi}{|Q|}$ and from the d-spacing a relative strain can be extracted $\epsilon_{(003)} = \frac{d-d_{ref}}{d_{ref}}$. Relative tilts and strain were calculated as compared to the mean value in the map.

Maps of the same AG region are shown for α, β, and (003) strain in Figure S1b, c, and d respectively. The striped domains are clearly observed in all images, with stripes visible in primarily the horizontal, but also the vertical direction. The average domain size, as extracted from the line cut in Figure S1e (marked by the dotted line in S1a), is 130 nm ± 10 nm, matching the expected spacing measured in PFM. Figure S1f schematizes relationship between α and β tilts with respect to the AG domain stripes. Unsurprisingly, the domain features are most pronounced in α as the ferroelastic domain stripes are formed due to the tilt mismatch between $r_4(r_2)$ and $r_3(r_1)$ variants. The line cut shows the difference in α tilt between domains to be 0.015° ± 0.005° and a smaller tilt of <0.004° is identified along the domain stripes (β). For 71° striped domains in BFO, the domains are typically

approximated to be fully coherent along the [010]pc direction but the observed tilt in β shows a tilt mismatch along this direction though it is ~25% of the [100] tilt. Beyond tilt, the ferroelastic domains present a strain variation of ~0.033%. Fig. S1e shows that strain is spatially offset from α tilt, this confirms expectations that strain comes from the tilt mismatch between r_4 and r_3, not from an inherent d-spacing difference between the two domain variants. Interestingly, β tilt is spatially aligned with strain (though reversed in magnitude).

The diffraction intensity in some regions of the film are lower, particularly in the regions between vertical and horizontal stripe domains (109° variant switches). In these regions, the Bragg condition is fulfilled (see Supplementary Note 5), however interference effects reduce the overall intensity. Additional interference effects may also be present due to 109° DWs, which would produce much higher frequency fringes, with oscillations larger than the Bragg peak width expected for 1-2 nm DWs.

While 3D strain mapping is more complete than 2D mapping, for large areas it is overly time consuming. Additionally, it was found that repetitive mapping of poled domain regions could act to depolarize the induced domain state, see Supplementary Note 6. However, repeated mapping of the AG domain region produced no noticeable changes in the domain structure.

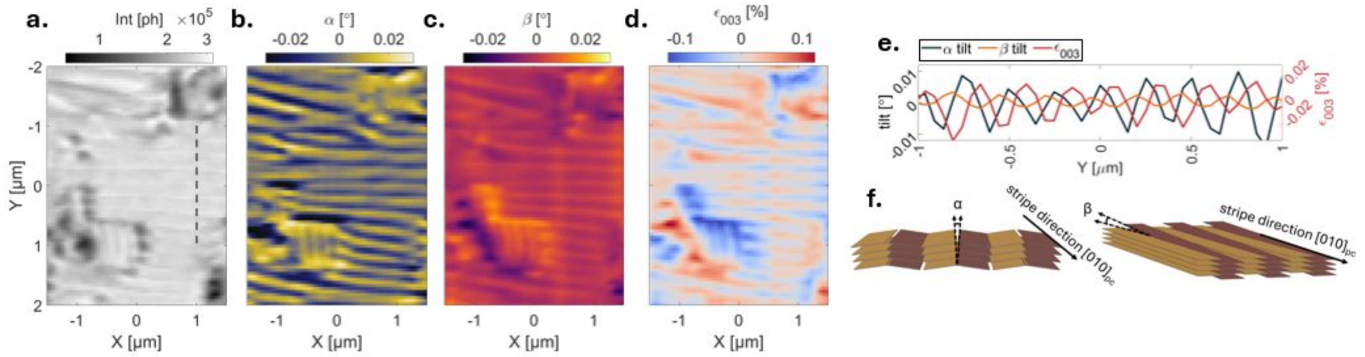

Figure S1: 3D strain mapping of AG region of the BFO film: (a) Integrated diffraction intensity, (b) α-tilt, (c) β-tilt, and (d) (003) strain. (e) Line cut extracted from dotted line in (a). (f) Schematic of α-tilt and β-tilt directions with respect to AG domains stripes.

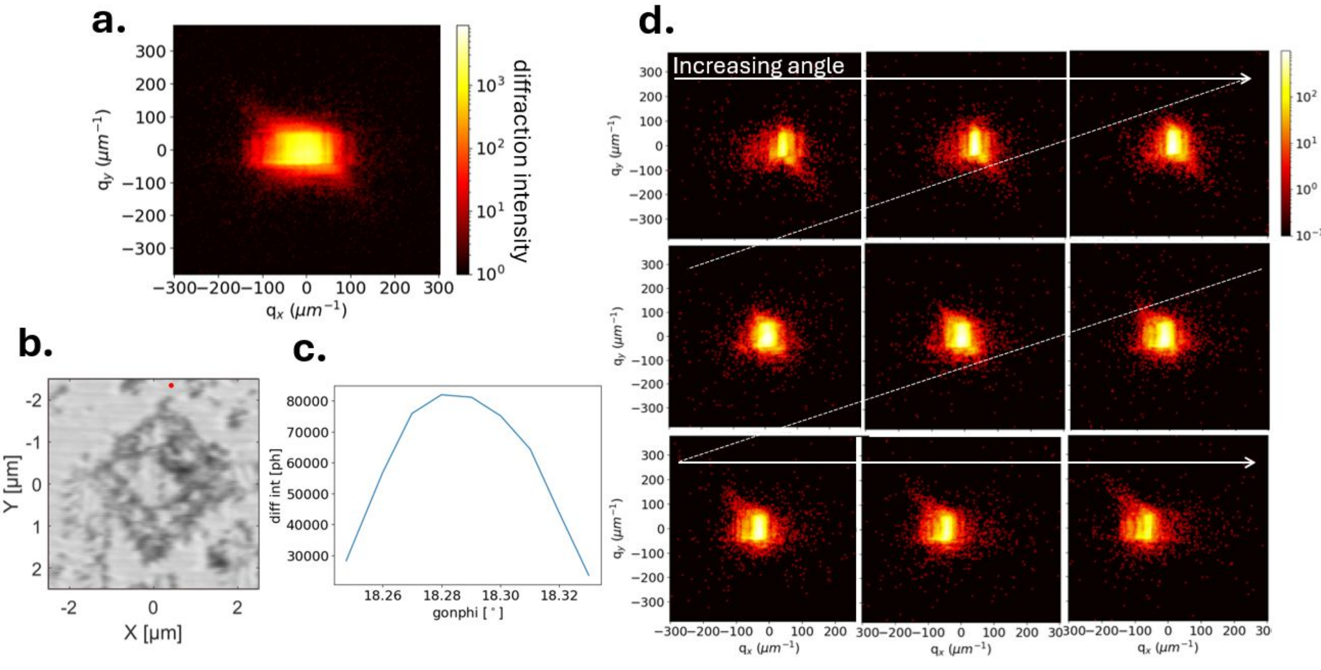

Figure S2: Example rocking curve from AG region of the BFO film. (a) Example Bragg peak extracted from the position marked by a red dot in (b). (c) Example rocking curve taken in the vicinity of the red dot in (b). (d) Extracted diffraction patterns along the rocking curve in (c).

Supplemental Note 2: Statistical analysis of nano-XRD tilt maps.

An additional BFO film, grown using the same conditions was measured with nano-XRD. Example maps for this second film are shown in Figure S3a with α-tilt and strain (004)pc shown on top and bottom respectively. Interestingly here, the ferroelastic domains are most pronounced when looking at the strain map. Here the striped domains are clearly visible in the AG region (-2.5 to 0 μm in Y) and a more disordered domain structure, with possibly more vertical stripes, is seen under the electrode (0 to 2.5 μm in Y).

A larger map was taken on this second BFO film to allow for statistical analysis of the real space α-tilt image. Figure S3b shows the larger area mapped and white dashed lines are used to outline the regions used for FFT analysis. These two regions were normalized and an FFT power spectrum was generated for each, the log power density is shown in Figure 1g. Figure S3b shows the normalized angular distribution of the power density. The AG regions shows primarily a sharp peak in the angular profile due to the strong ordering of the horizontal ferroelastic domains. However, for the electrode region, the power spread is much flatter and broader, this is indicative of a much more disordered domain structure present under the electrode. While FFT analysis is not possible on electrode:0V (Figure 1e) due to insufficient statistical area, it is possible to compare the distribution of α-tilt angles. This is shown as a histogram in Figure S3c. Here it is seen that the electrode region has a smaller spread of angles (narrower distribution) which may be related to the increased disorder in the electrode region, where the individual ferroelastic domains are not as well defined.

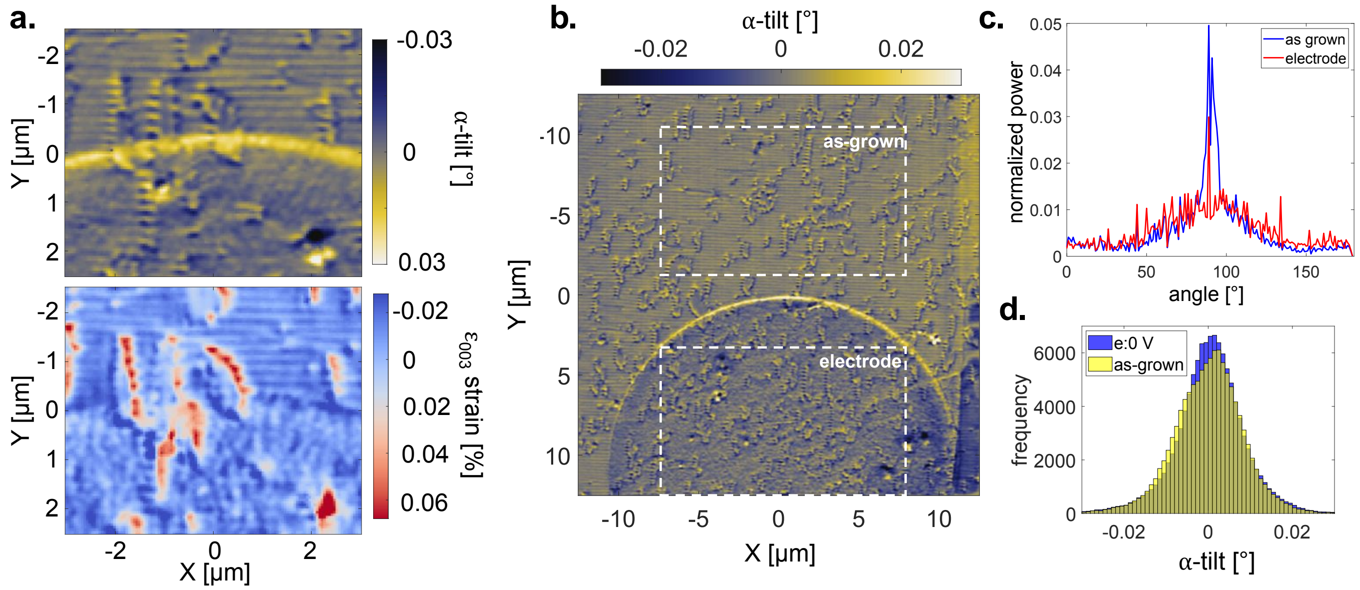

Figure S3: Additional analysis of domain tilts in AG and buried BFO. (a) Example tilt (top) and strain (bottom) maps of second BFO film (b) Large area mapped for a comparable statistics on the AG region and electrode region. White dotted lines show regions used for 2D FFT analysis. (c) Angular FFT profiles extracted from 2D FFT magnitude images in Figure 1g. (d) Histogram of α-tilt angular distribution for pristine electrode:0V compared to the surrounding AG region.

Supplementary Note 3: Determining strain without 3D rocking curves.

As described in Supplementary Note 1, true quantification of tilt and strain requires mapping diffraction patterns in 3D as strain is the modulus of the peak position in all three dimensions, q_x, q_y, q_z, and tilt has components in multiple q directions as well. However, instead of taking full 3D rocking curves (reciprocal space maps) at each position, a good approximation can be to find the angle at which the Bragg peak maximum occurs for each position, and then find the 2D COM on the detector to calculate the tilt and d-spacing change. In this approach, the COM of $q_1(\sim q_x)$ corresponds to d-spacing change and q_2 (q_y) to α-tilt. In other words, finding the θ angle (at each sample position) in which the diffraction intensity is maximized allows for the calculation of strain and α-tilt from a singular 2D diffraction pattern at each sample position.

In the case of mapping electrodes e:0V and e:6V in Figure 2, a rocking curve was taken in the uniform, AG region of the film, shown in Figure S2. The maximum angle was then used for the 2D mapping of e:0V and e:6V electrodes. While angular optimization was not done on the entire mapped region, it can be seen from the diffraction intensity maps (Figure 2d), that the scattering intensity does not vary significantly between the AG region and the electrode region – excluding regions in which further disorder occurs such as switching from horizontal to vertical domain stripes. This indicates that the Bragg condition is fulfilled both in the AG region and under the electrodes, within the angular step size used (0.05°). As such, it is appropriate to approximate 2D COM changes in q_x as strain variations and q_y as α-tilt variations.

Supplementary Note 4: Additional biased electrodes.

Figure S4 shows full maps of all electrodes for α-tilt (top) and q_x (bottom). The most obvious changes, both for α and q_x, are observed for e:6V and e:8V. This includes a more pronounced higher α angle ring around the electrode and a larger difference in q_x magnitude between the AG and biased regions. Electrode e2:6V looks more similar to the unbiased electrode e:0V, though there is a slightly stronger contrast in q_x. This is possibly because PUND measurements were not performed on e2:6V, it was instead polarized using direct bias from a PFM tip at a lower frequency (2 Hz) compared to the PUND biased e:6V and e:8V (5 kHz).

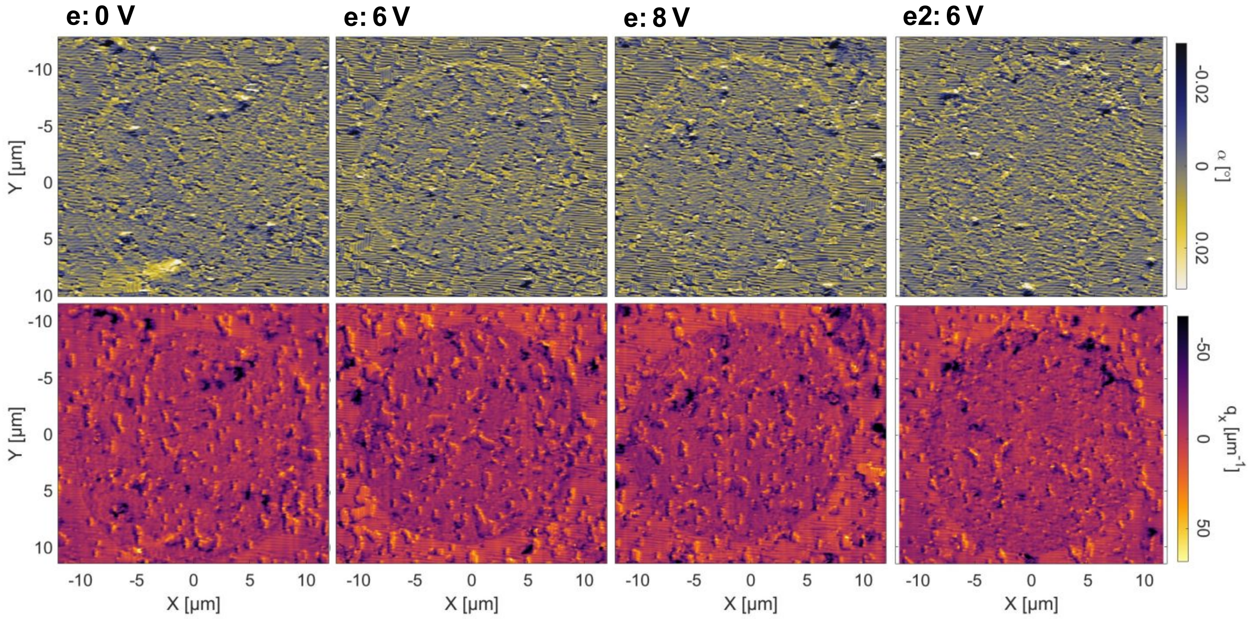

Figure S4: Full α-tilt (top) and q_x (bottom) maps for all measured electrodes.

Supplementary Note 5: Interference effects due to domain walls.

Though diffraction intensity is lower in the regions between vertical and horizontal stripe domains (109° variant switches), the decreased intensity is likely from interference effects, opposed to being off of the Bragg condition. Figure S5a shows an example diffraction pattern from the 109° variant region, as marked by the blue dot in Figure S5b. Here we can observe interference effects, which could result from being in the vicinity of vertical domain stripes. Measuring a number of angles in the vicinity of this 109° variant, we cannot observe a clear rocking curve. However, looking at the individual diffraction patterns in Figure S5d, we do observe a mostly symmetric transition from fringes on the left of the peak to the right of the peak. Indeed, the Bragg maximum is defined not by the angle of maximum intensity, but instead as the angle at which the symmetric centre of the Bragg peak is observed (though these are usually equivalent). This indicates that the Bragg maximum has likely been fulfilled in this angular range despite the lack of clear intensity maximum. Additionally, particularly for the fourth pattern, we see further interference effects that could result from 109° DWs, which would produce much higher frequency fringes, resulting in the drop in intensity of the Bragg peak.

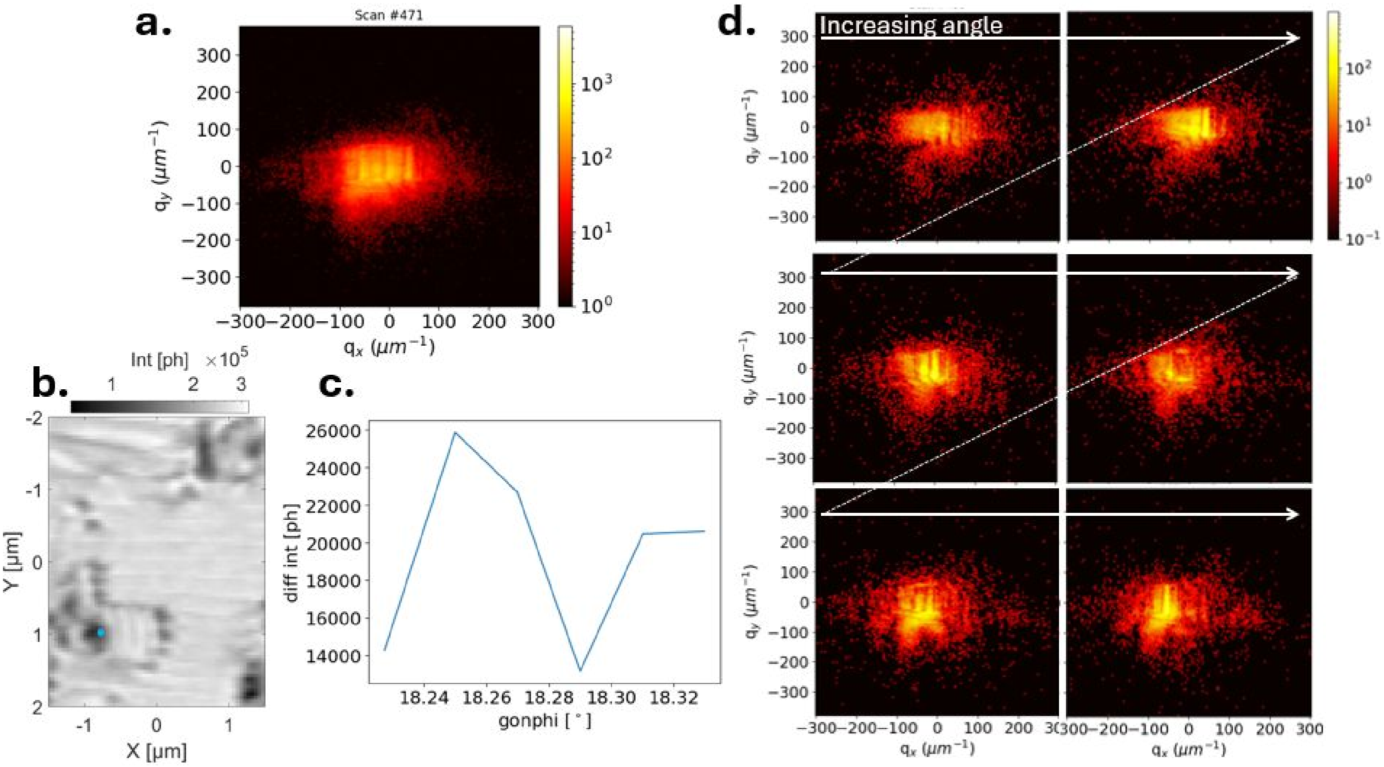

Figure S5: Example rocking curve from the 109° variant region of the BFO film. (a) Example Bragg peak extracted from the position marked by a blue dot in (b). (c) Example rocking curve taken in the vicinity of the blue dot in (b). (d) Extracted diffraction patterns along the rocking curve in (c).

The same interference effects are observed for the p-up polarized region of the box-in-box structure. Here a significantly lower intensity is observed as compared to the AG and the p-down regions. Figure S6a shows an example diffraction pattern, where significant interference is clearly observed as compared to the AG region in Figure S1a. This is taken in the p-up region marked by the red dot in Figure S6b. A rocking curve in the vicinity is shown in Figure S6c. Though there are some variations in intensity, a peak is observable, suggesting that here the Bragg condition is fulfilled, however interference effects result in the observed intensity reduction. For additional details, Figure

S6d shows the diffraction patterns in the region across the rocking curve. These show a mostly symmetric effect with fringes transitioning from left to right of the primary peak with increasing angles.

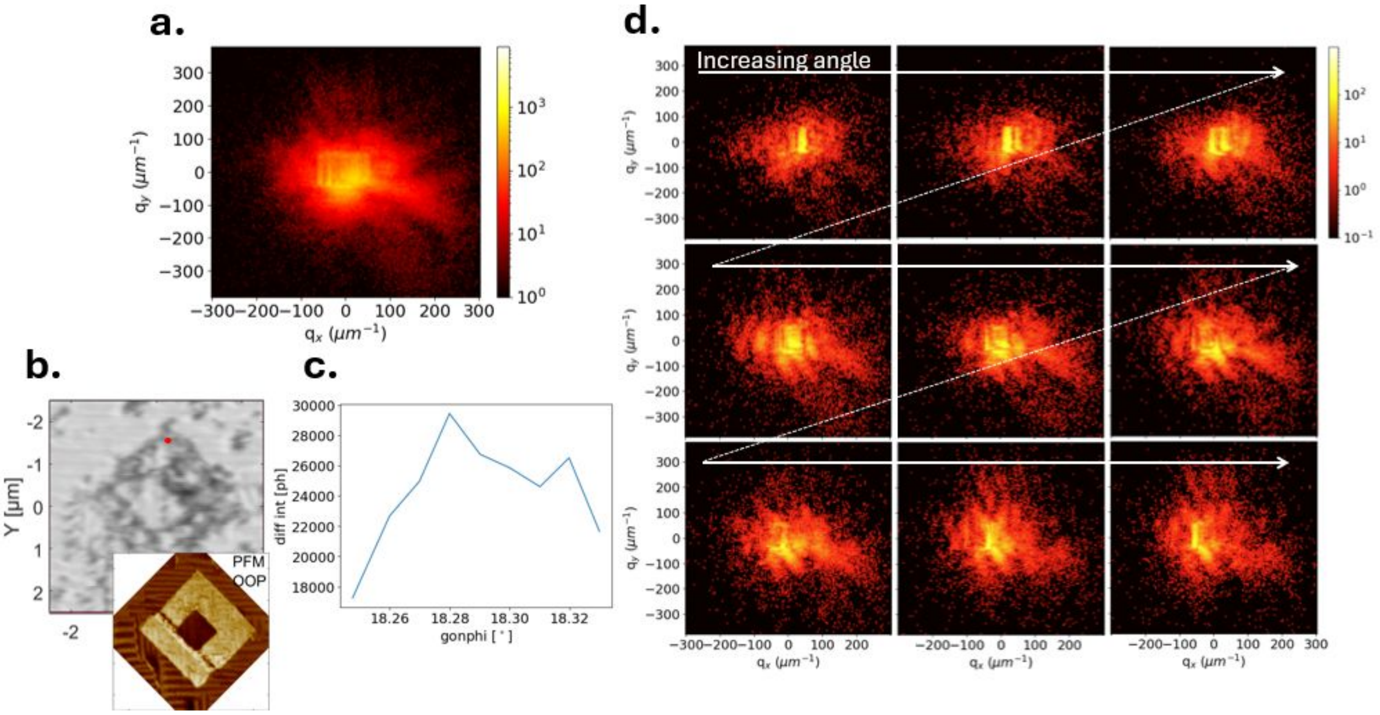

Figure S6: Example rocking curve from the p-up region of the box-in-box polarized region of BFO. (a) Example Bragg peak extracted from the position marked by a red dot in (b). (c) Example rocking curve taken in the vicinity of the red dot in (b). (d) Extracted diffraction patterns along the rocking curve in (c).

Supplementary Note 6: Additional box-in-box structure and beam damage testing.

An additional box-in-box region (2) was characterized and presented comparable results to the box-in-box 1 structure. Figure S7a shows the out-of-plane polarization measured by PFM. Results from the parallel and perpendicular scattering for box-in-box 2 are shown in (b) and (c) respectively. The top map shows the integrated diffraction intensity, center shows the q_y COM, and lower shows q_x COM.

After measuring the box-in-box region 4-5 times, the difference between the AG region and the polarized region is significantly reduced as seen by the maps in Figure S7d. This indicates that repetitive exposure to the X-ray beam can act to depolarize the film.

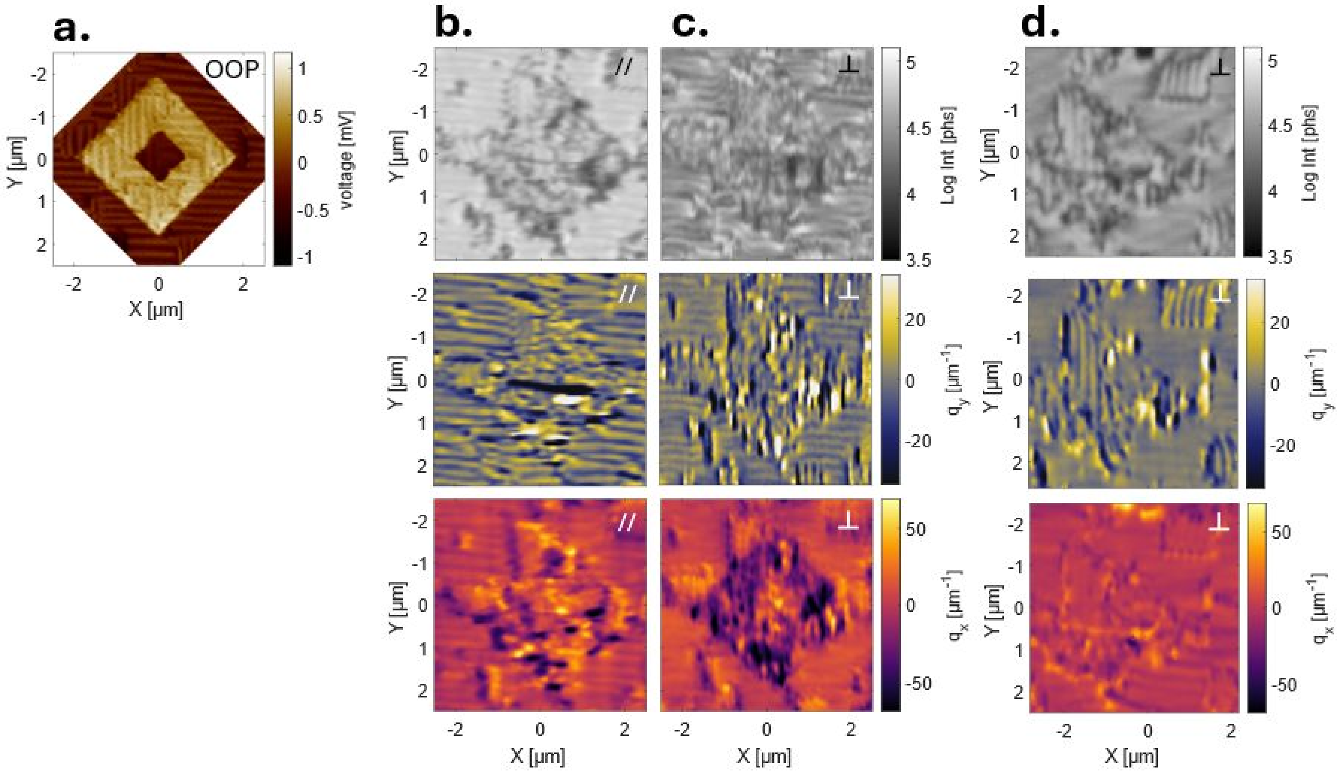

Figure S7: Box-in-box 2 structure and beam damage evaluation. (a) Out-of-plane PFM map of an additional box-in-box structure. (b) Parallel and (c) perpendicular scattering results. Top shows diffraction intensity, center shows q_y COM, and bottom shows q_x COM. (d) Map of box-in-box 2 after repeated measurements.

Though the X-ray beam appears to have an effect on the polarization of the film, it does not appear to easily damage the AG domain structure. This is shown by the repeated maps measured in the AG region of Figure S8.

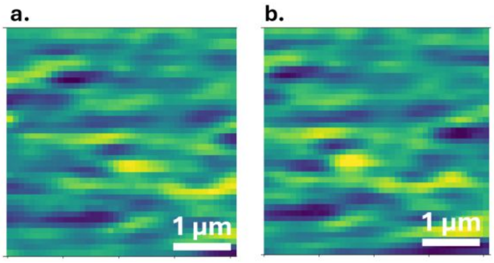

Figure S8: Beam damage test for AG region of BFO. Integrated diffraction intensity maps after a single exposure (a) and after 5 repetitions (b). The diffraction intensity is unchanged between the two maps despite a long exposure (0.1 s per point) and high flux (7e9 ph/s).

Supplementary Note 7: Supplementary Methods

XRD measurements: Diffraction measurements were conducted using the Diffraction Endstation of the NanoMAX beamline at MAX IV Laboratory. [23] Symmetric scattering, as shown in Figure 1b, was used to probe the BFO (003)pc Bragg condition. Measurements were taken at 15 keV and an incident angle of 18°, producing a beam footprint of ~60 nm x 180 nm. The sample was mounted with the majority of ferroelectric stripes parallel to the beam direction (along DSO [001]) to maximize spatial resolution. Nano-XRD was performed, collecting high resolution 2D diffraction patterns of the (003)pc peak, as exampled in Figure 1c, at each probe position while scanning the sample under

the X-ray beam with a 50 nm step size (example by the white dotted grid in Figure 1b). The center of mass of the Bragg peak was calculated for each probe position, producing a tilt map in Figure 1e. See Supplementary Note 1 for more details on COM and tilt determination. [24,25] Tilt maps shown here are produced from diffraction taken at a single angle instead of from a full 3D angular dataset, therefore they are only approximate (see Supplementary Note 3).

Sample fabrication: The BFO/SRO films were grown on single-crystalline$(110)_o$-oriented DSO substrates (CrysTec GmbH) by pulsed laser deposition using a 248 nm KrF excimer laser. The SRO buffer layer was deposited at 700°C under 0.016 mbar oxygen partial pressure with a laser fluence of 1.35 J cm^{-2} and a laser repetition rate of 2 Hz. The BFO films were subsequently grown at 680°C under 0.12 mbar oxygen partial pressure with a laser fluence of 1.71 J cm^{-2} and a laser repetition rate of 8 Hz. After the cooling process, the films were transferred into the DC-magnetron sputtering chamber at a base pressure of ~10^{-8} mbar. The top 100 nm Pt layer was deposited under an argon pressure of 10^{-3} mbar. The circular electrodes with 20 µm diameter were patterned by photolithography and argon plasma etching.

Electrical poling: The electrodes were biased with a home-built ferroelectric test system with a probe station and a PFM tip. The positive-up-negative-down (PUND) technique was used for two electrodes (e:6V, e:8V). A sequence of pulses was applied to the Pt top electrodes at 5 kHz. Firstly, a preset voltage pulse of negative polarity sets the polarization, and subsequently, two pulses of positive polarity were applied, followed by two pulses of opposing polarity. Electrode e2:6V was biased using a PFM tip at 2 Hz with -6V. The pulses were applied to the top electrode via the probe and PFM tip, and the bottom SRO electrode was grounded. No biasing was performed during the X-ray measurements, only ex-situ.

PFM poling and measurements: The PFM measurements and the electric-field tip poling were performed using a Bruker Multimode 8 atomic force microscope with µmasch HQ:NSC35/Pt tips in contact mode. During raster-scanning, a 2.5 V peak-to-peak AC voltage modulation was applied to the tip at 15 kHz. Ferroelectric poling was induced by applying a DC bias of ±5 V to the tip. The bottom SRO electrode was grounded.
